# Detection of Extended-Spectrum β-Lactamases, Metallo-β-Lactamases, Antimicrobial Resistance Profiles, and Biofilm-Forming Capacity in *Pseudomonas aeruginosa* Strains Recovered From Dogs With Otitis Externa in Italy

**DOI:** 10.1155/vmi/5566151

**Published:** 2025-05-16

**Authors:** Francesca Paola Nocera, Adriana Chiaromonte, Rossana Schena, Francesca Pizzano, Sinem Arslan, Chiara Pedicini, Luisa De Martino

**Affiliations:** Department of Veterinary Medicine and Animal Production, University of Naples Federico II, Naples 80137, Italy

**Keywords:** antimicrobial resistance, biofilm formation, canine otitis externa, ESBL and MBL genes, *Pseudomonas aeruginosa*

## Abstract

*Pseudomonas aeruginosa* is considered the second major causative agent of otitis externa in dogs, after *Staphylococcus pseudintermedius*. This study aimed to evaluate the antimicrobial resistance profiles and to detect the extended-spectrum β-lactamase (ESBL) and metallo-β-lactamase (MBL) genes in *Pseudomonas aeruginosa* (*P. aeruginosa*). Precisely, seventeen *P. aeruginosa* strains, recovered from auricular specimens of dogs affected by otitis externa, were identified by MALDI-TOF MS. Antimicrobial susceptibility testing was carried out against eleven clinically relevant antimicrobials using the Kirby Bauer disk diffusion method on Mueller Hinton agar plates. The PCR assay was performed to detect ESBL *bla*_*CTX*−*M*_,  *bla*_*TEM*_,  *bla*_*SHV*_,  *bla*_*PER*_, and MBL *bla*_*IMP*_,  *bla*_*OXA*−48_,  *bla*_*VIM*_,  *bla*_*NDM*_,  *bla*_*GES*_ genes. The results showed that *P. aeruginosa* isolates had a phenotypic resistance value of 100% to ceftazidime, imipenem, and meropenem, followed by piperacillin-tazobactam, sulfamethoxazole-trimethoprim (94%), and aztreonam (88%). An alarming result was represented by the high prevalence of multidrug-resistant strains with 100% of the total isolates. The most common ESBL-genotype combination was *bla*_*PER*_ + *bla*_*SHV*_ (29.4%). Thirteen isolates (76.5%) carried together *bla*_*VIM*_ + *bla*_*GES*_ genes, which resulted to be the most common MBL-genotype combination. All the isolates harboring ESBL and MBL genes were biofilm producers, evaluated by the crystal violet-based assay and PCR. Precisely, 76.5% were strong biofilm producers, and 23.5% resulted in being moderate producers. No relationship was observed between strong or moderate biofilm producers and numerical variability of ESBL and MBL genes. This study revealed worrying antimicrobial resistance profiles of *P. aeruginosa-*associated canine otitis externa, considering also the zoonotic potential of this pathogen.

## 1. Introduction

Otitis externa, an inflammation of the external ear canal, is a major burden in the canine population, affecting dogs of any gender and age, with a prevalence rate up to 20% [1]. This skin disorder has a multifactorial etiology, and primary factors (i.e., allergies, the presence of foreign bodies, adverse food reactions, and keratinization disorders) often create optimal conditions for growth and proliferation of bacteria and yeasts in the ear canal [2].

In dogs, bacterial otitis externa is mainly caused by *Staphylococcus pseudintermedius*, an opportunistic pathogen resident on canine skin and mucous membranes [3–5]. *Pseudomonas aeruginosa* (*P. aeruginosa*) is considered the second major causative agent of canine otitis externa globally [4, 6]. Currently, *P. aeruginosa* has become a challenging and worrisome pathogen in veterinary medicine, since it is often associated with chronic and recurrent otitis externa in pet animals, due to its notorious ability to form robust biofilms on various biotic and abiotic surfaces and its intrinsic and acquired antimicrobial resistance [7]. Notably, *P. aeruginosa* belongs to the ESKAPE group together with *Enterococcus faecium*, *Staphylococcus aureus*, *Klebsiella pneumoniae*, *Acinetobacter baumannii*, and *Enterobacter* spp; six highly virulent bacterial pathogens able to ‘escape' commonly used antimicrobials for their troubling multidrug-resistant (MDR) profiles [8]. Intriguingly, in recent years, the growing prevalence in human medicine of life-threatening nosocomial infections by MDR *P. aeruginosa* has been related to the reduced choice of effective antimicrobials to be selected, which have led to the search for a multifaceted antibiotic resistance strategy as combination therapies, including conventional antibiotics and/or alternative therapeutic treatments [9]. Clinical *P. aeruginosa* isolates resistant not only to aminoglycosides, β-lactams, and fluoroquinolones but also to third and fourth generations cephalosporins and carbapenems and to the recently formulated ceftolozane-tazobactam and ceftazidime-avibactam drugs have been increasingly recovered, thus becoming one of the greatest challenges in human and veterinary clinical practice worldwide [6, 10–12]. Many mechanisms are involved in *P. aeruginosa* resistance to antimicrobials such as overexpression of efflux pumps, reduction of absorption, and production of β-lactamases, which are categorized in extended-spectrum β-lactamases (ESBLs) and metallo-β-lactamases (MBLs) [7]. The production of β-lactamases is considered the main acquired antimicrobial resistance mechanism in Gram-negative bacteria, displaying resistance to antimicrobials belonging to the family of β-lactams [13].

ESBLs are a group of β-lactamases, frequently plasmid-encoded, responsible for bacterial resistance to penicillins, cephalosporins (first-, second-, and third-generation), and monobactams (aztreonam), by hydrolyzing these antimicrobials. These enzymes are known to be susceptible to β-lactamase inhibitors, such as clavulanic acid, sulbactam, and tazobactam [14].

MBLs represent the most resilient β-lactamases, whose expression can be mediated by both chromosomal and plasmid genes [15]. MBL enzymes are not only able to hydrolyze carbapenems such as imipenem and meropenem but also other antibiotics belonging to the β-lactam family, thus showing the widest spectrum of activity among β-lactamases [16]. In particular, the emergence and dissemination of these enzymes have been a consequence of an increased administration of carbapenems to treat infection caused by ESBL producing Gram-negative bacteria, leading to new challenge of carbapenem-resistant bacteria [17]. Different from ESBLs, MBLs are not susceptible to β-lactamase inhibitors, making ineffective carbapenems, which are considered critically important antibiotics administered for the treatment of severe infections. Furthermore, MBL-producing bacterial pathogens often show MDR profiles [16], promoting their circulation and widespread in hospitals, community settings, and the environment [18]. In addition, from the last few years, pet animals have been regarded as reservoirs of antimicrobial-resistant bacteria for humans, due to the overuse or misuse of antimicrobials also in veterinary medicine [18], so that close contact among pets and households might play an important role in the transmission of antimicrobial-resistant bacteria and genetic elements [19]. Currently, information about the spread of ESBL and MBL *P. aeruginosa* producing isolates in pet animals is still scarce. Indeed, nowadays, the susceptibility to these antibiotics is rarely evaluated, due to the ban on the use of carbapenems, monobactams, and combination of cephalosporins with β-lactam inhibitors in European countries as established by the European Union with the recent Regulation containing the list defining a list of antimicrobials authorized solely for human use (Commission Implementing Regulation EU 2022/1255) [20], making them unavailable as therapeutic options for life-threatening MDR ESBL- and MBL-producing *P. aeruginosa* infections in animals. Previously, these antimicrobial classes including also penicillins (carboxy penicillins and ureido penicillins) with β-lactam inhibitors were already indicated as “critically important antimicrobials” by the World Health Organization [21]. In addition, according to the Antimicrobial Advice Ad Hoc Expert Group (AMEG) categorization of the antibiotics for use on animals, they belong to category A (“Avoid”), so their off-label administration is allowed in companion animals only under exceptional circumstances [22].

For this reason, studies on the detection of ESBLs and MBLs as *P. aeruginosa* positive isolates are needed to control infections and to prevent their dissemination in veterinary settings.

The aim of this study was to evaluate the phenotypic and genotypic antimicrobial resistance profiles and, precisely, the detection of the ESBL and MBL genes in clinical strains of *P. aeruginosa* associated with canine otitis externa in Italy. Moreover, since it is well known that biofilm may significantly drive antibiotic resistance, this study focused also on the biofilm formation evaluation and the related gene detection.

## 2. Materials and Methods

### 2.1. Specimen Collection

During the year 2023, *P. aeruginosa* strains were recovered from routine bacteriological examinations of canine samples. The specimens consisted of 60 auricular swabs collected from dogs suffering from unilateral or bilateral chronic otitis, referred to the University Veterinary Teaching Hospital, Department of Veterinary Medicine and Animal Production (University of Naples “Federico II,” Italy). Auricular sampling was performed by carefully inserting and gently rotating a sterile cotton swab within the ear canal to collect some discharge or debris from the canal walls. For cases of bilateral otitis, separated swabs were used for each affected ear. Each swab was then immediately placed into a Stuart W/O CH transport medium (Aptaca Spa, Asti, Italy) and transferred to the Bacteriology laboratory within 2 h.

### 2.2. Isolation and Identification of *Pseudomonas aeruginosa* Strains

Once delivered at the Bacteriology laboratory of the above-mentioned Department, the auricular swabs were streaked on different solid agar media: Columbia CNA agar with 5% sheep blood for the recovery of Gram-positive bacteria, Mac Conkey agar (MCA) for the selection of Gram-negative bacteria, Mannitol Salt agar for the selective growth of staphylococci, and Sabouraud dextrose agar for yeast isolation. All plates were incubated aerobically at 37°C for 24–48 h. Precisely, *P. aeruginosa* isolation was performed by using MCA agar plates (Liofilchelm, Teramo, Italy), a selective and differential medium for Gram-negative bacteria. The suspected *Pseudomonas* spp.-recovered colonies were preliminary examined for their morphology, pigment production, and the absence of lactose fermentation on MCA and then further screened by Gram's staining and the oxidase test. The identification was assessed on fresh colonies by matrix-assisted laser desorption ionization time-of-flight mass spectrometry (MALDI-TOF MS) analysis by use of the MALDI Biotyper sirius system (Bruker Daltonics Inc., Bremen, Germany). The identification was based on the score values released by the manufacturer's instructions. Precisely, according to Bruker biotyper's recommendations, a score > 2.0 indicates a “highly probable species identification,” a score from 1.70 < *x* < 1.99 indicates a “probable genus identification,” and a score < 1.70 indicates an “unreliable identification.” *Pseudomonas aeruginosa* ATCC 27853 was used as the quality control strain.

Pure *P. aeruginosa* colonies were stored in Microbank tubes at −80°C (Pro-Lab Diagnostics Inc., Round Rock, TX, USA) for further investigations.

### 2.3. Antibiotyping of Canine *Pseudomonas aeruginosa* Strains

The antimicrobial resistance profile of all strains was evaluated by the Kirby Bauer disc diffusion method on Mueller Hinton agar plates (Liofilchem, Teramo, Italy), accomplishing the European Committee on Antimicrobial Susceptibility Testing guidelines [23]. The following panel of 11 antimicrobials was tested: amikacin (AK-30 μg), aztreonam (ATM-30 μg), ceftazidime (CAZ-30 μg), ciprofloxacin (CIP-30 μg), gentamicin (CN-10 μg), imipenem (IMI-10 μg), marbofloxacin (MAR-5 μg), meropenem (MRP-10 μg), piperacillin-tazobactam (TPZ-110 μg), sulfamethoxazole-trimethoprim (SXT-25 μg), and tobramycin (TOB-10 μg), belonging to 7 antimicrobial classes: aminoglycosides, carbapenems, extended-spectrum cephalosporins (III generation), fluoroquinolones, monobactams, penicillins + beta-lactamase inhibitors, and sulfonamides. Strains showing resistance to at least one antimicrobial of ≥ 3 antimicrobial classes were categorized as MDR strains according to Magiorakos et al. [24].

The choice of antimicrobials was based on those frequently used in small animal practice for ear infections. Critically important human antimicrobials not authorized for veterinary use (imipenem, meropenem, aztreonam, and piperacillin-tazobactam) were also tested to track resistance in the animal strains.

The reference strains *Pseudomonas aeruginosa* ATCC 27853 served as quality control for antimicrobial susceptibility testing.

### 2.4. Molecular ESBL and MBL Gene Detection in Canine *Pseudomonas aeruginosa* Strains

DNA extraction was carried out from isolated *P. aeruginosa* colonies grown on MCA agar plates by using the commercial QIAamp DNA Mini Kit (QIAGEN GmbH, Hilden, Germany) following the manufacturer's instructions. The bacterial DNA was stored at −20°C for further studies.

The presence of ESBL *bla*_*CTX*−*M*,_ *bla*_*TEM*_,  *bla*_*SHV*_,  *bla*_*PER*_, and MBL *bla*_*OXA*−48_,  *bla*_*IMP*_,  *bla*_*VIM*_, *bla*_*NDM*_,  *bla*_*GES*_ genes was detected in collected *P. aeruginosa* strains by using specific primers, synthetized by Eurofins Genomics GmbH (Ebersberg, Germany). *Pseudomonas aeruginosa* ATCC 27853 was used as a negative control strain for ESBL and MBL genes. Primer sequences, amplicon sizes, and annealing temperatures for each tested gene are reported in Table 1.

The amplified products were analyzed by gel electrophoresis. Precisely, PCR products were then separated by electrophoresis in 1.5% agarose gels with 1xTBE buffer. Electrophoresed gels were visualized and photographed under UV illumination.

### 2.5. Crystal Violet-Based Biofilm Assay and Detection of Biofilm-Encoding Genes in *Pseudomonas aeruginosa* Strains

The crystal violet quantitative assay was carried out to investigate *P. aeruginosa* strains' biofilm production ability, according to Stepanovic et al. [32] with some modifications. Overnight cultures of *P. aeruginosa* were adjusted to OD_600nm_ of 0.2 (∼10^8^ CFU/mL) with fresh brain heart infusion (BHI) broth medium (Liofilchem, Teramo, Italy). Standardized bacterial suspensions were diluted 1:2 in 200 μL of BHI broth and inoculated into the sterile wells of the flat-bottomed 96-well polystyrene microplate (Corning, Inc., New York, USA). BHI broth medium was used as a negative control to check the sterility of media and to determine background optical density (OD). After the incubation at 37°C for 24 h, wells were gently washed three times with sterile phosphate buffered saline (PBS) to remove free floating planktonic bacteria and air-dried. Biofilms were stained with 200 μL of 1% crystal violet (BioMérieux, Marcy l'Etoile, France) for 30 min at room temperature and then washed again with sterile water to remove excess dye. After air-drying, crystal violet was eluted by incubating statically with 100% ethanol for 20 min at room temperature. The absorbance of the stained biofilms, expressed in OD values, was measured at 570 nm with a multiwell reader (Multiskan FC, Thermo Fisher Scientific, Milan, Italy). All experiments were performed in triplicate and repeated three times. In addition, according to Stepanovic et al. [32], the cut-of value (ODc), defined as three standard deviations (SDs) above the mean OD of the negative control, was established, and the strains were classified as follows: nonbiofilm producer (OD < ODc), weak biofilm producers (ODc < OD < 2 × ODc), moderate biofilm producers (2 × ODc < OD < 4 × ODc), and strong biofilm producers (4 × ODc < OD).

In addition, the extracted DNA of all *P. aeruginosa* strains was investigated for the presence of the biofilm-encoding genes *alg*D, *psl*D, and *pel*F by PCR, using specific primers [33].

Primer sequences, amplicon sizes, and the amplification program for the aforementioned biofilm-encoding genes are shown in Table 2.

### 2.6. Data Analysis

All diagnostic results generated by the microbiological diagnostic laboratory were recorded and entered into a Microsoft 365 Excel spreadsheet for successive analysis. Descriptive statistical analysis was employed to analyze the prevalence of *P. aeruginosa* and the frequencies of antimicrobial resistance among recovered isolates. The reported data relating to biofilm formation are presented as mean values of three independent experiments ± SD, and the graph was created by using Sigma Plot software (Jandl, Erkrath, Germany). Furthermore, two-tailed Fisher's exact test was performed on the relationship between phenotypic biofilm production ability and biofilm genotype, as well as between ESBL/MBL and biofilm gene detection using the Statistical Program Easy Fisher Exact Test Calculator. A *p* value < 0.05 was considered as statistically significant.

## 3. Results

### 3.1. *Pseudomonas aeruginosa* Isolation and MALDI-TOF MS Identification

During the year 2023, a total of 60 auricular swabs were collected from dogs affected by otitis externa. The frequency of isolation of *P. aeruginosa* was 28.3%, and the 17 strains were recovered from the ear specimens of dogs belonging to the following breeds: 8 mixed-breed dogs, 2 Cocker Spaniel, 3 German Shepherds, 2 Golden Retrievers, and 2 French Bulldogs. Precisely, the suspected 17 *P. aeruginosa* strains were all detected in monoculture on MCA agar plates, and some of them turned the medium color, after incubation, to greenish for the production of pigments such as pyocyanin and pyoverdine. Furthermore, all the recovered strains appeared to be rod-shaped Gram-negative bacteria on the smears, resulting to be also catalase and oxidase positive. In addition, all isolated *P. aeruginosa* strains were identified by MALDI-TOF MS with a log(score) between 2.3 and 2.6, indicative of a highly probable species identification according to the manufacturer's guidelines.

### 3.2. Phenotypic Antimicrobial Resistance Profiles of *Pseudomonas aeruginosa* Strains

**Figure 1:**
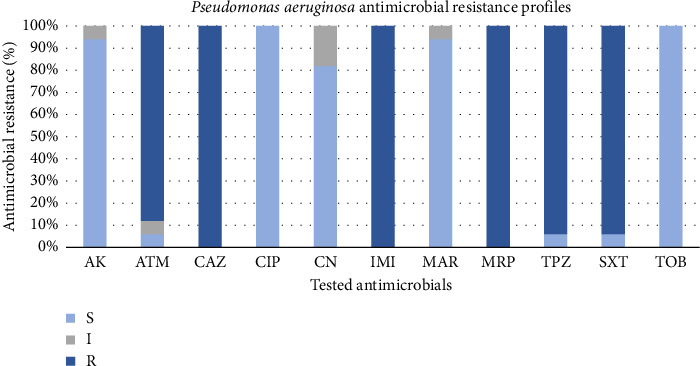
Antimicrobial resistance profiles of 17 *Pseudomonas aeruginosa* strains. The strains were classified as susceptible (S), intermediate (I), or resistant (R). Abbreviations: AK = amikacin, ATM = aztreonam, CAZ = ceftazidime, CIP = ciprofloxacin, CN = gentamicin, IMI = imipenem, MAR = marbofloxacin, MRP = meropenem, TPZ = piperacillin-tazobactam, SXT = sulfamethoxazole-trimethoprim, and TOB = tobramycin.

The phenotypic antimicrobial resistance profiles of the recovered *P. aeruginosa* strains are reported in Figure 1. *P. aeruginosa* isolates had a resistance value of 100% (17/17 strains) to ceftazidime, imipenem, and meropenem, followed by piperacillin-tazobactam and sulfamethoxazole-trimethoprim with a value of 94% (16/17 strains) for both antimicrobials and aztreonam (88%; 15/17 strains), indicating the possible presence of ESBL and MBL enzymes.

The highest susceptibility level equal to 100% (17/17) was recorded for both ciprofloxacin and tobramycin, followed by amikacin and marbofloxacin both with a value of 94% (16/17 strains) and by gentamicin (82%; 14/17 strains).

An alarming result was that all the surveyed *P. aeruginosa* appeared to be MDR strains, showing resistance to at least 1 agent in ≥ 3 antimicrobial classes.

### 3.3. Genomic Detection of ESBLs and MBLs in *Pseudomonas aeruginosa* Strains

The ESBL genotypic resistance was driven by *bla*_*PER*_ (100%; 17/17), followed by *bla*_*SHV*_ (29.4%; 5/17), *bla*_*TEM*_ (23.5%; 4/17), and lastly by *bla*_*CTX*−*M*_ (17.6%; 3/17). The most common ESBL-genotype combination was *bla*_*PER*_ + *bla*_*SHV*_ (29.4%).

Referring to MBL-genotypic resistance, *bla*_*VIM*_ was detected in all 17 *P. aeruginosa* isolates (100%), followed by *bla*_*GES*_ (76.5%; 13/17), *bla*_*OXA*−48_ and *bla*_*NDM*_ found both in 23.5% of the strains (4/17) and lastly *bla*_*IMP*_ (17.6%; 3/17). Thirteen isolates (76.5%) carried together *bla*_*VIM*_ + *bla*_*GES*_ genes, which resulted to be the most common combination. Furthermore, the simultaneous presence of all five MBLs *bla*_*IMP*_,  *bla*_*OXA*−48_,  *bla*_*VIM*_,  *bla*_*NDM*_,  and *bla*_*GES*_ was detected in two strains (11.8%).

Interestingly, the obtained results displayed a concordance between ESBL and MBL phenotype and genotype among the screened *P. aeruginosa* strains, as reported in Table 3.

The PCR results for the detection of *bla*_*SHV*_,  *bla*_*TEM*_,  and *bla*_*GES*_ genes are reported in Figures S1, S2, and S3.

### 3.4. Determination of Biofilm-Forming Capacity and Biofilm Associated-Gene Detection

**Figure 2:**
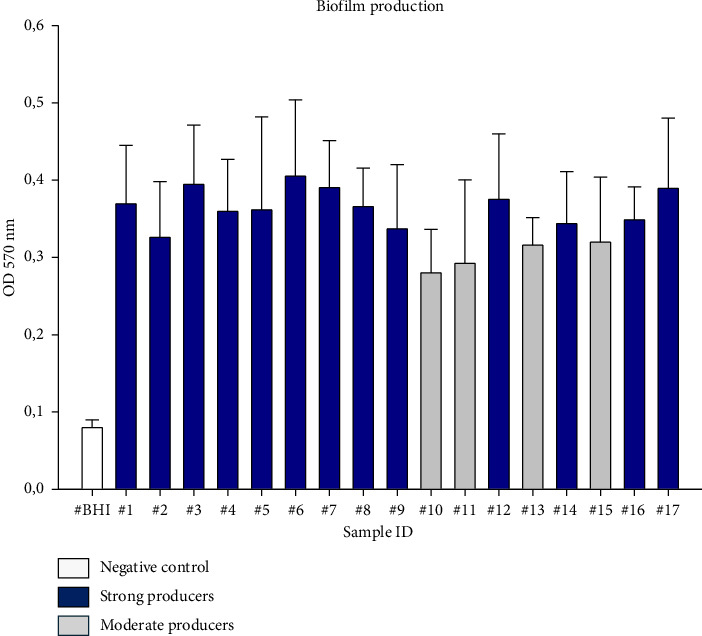
Biofilm-producing ability of the 17 *Pseudomonas aeruginosa* strains on the flat-bottomed 96-well polystyrene microplate. Biofilms were formed in BHI at 37°C for 24 h, and biofilm production ability was measured by the crystal violet quantitative assay. BHI broth medium was used as a negative control. Biofilm production ability was determined using the mean OD570 of the negative control (0.08), values between 0.08 and 0.16 (2 × the negative control value of 0.08) were considered to be strains that were weak producers (0%), values between 0.16 and 0.32 (4 × the negative control value of 0.08) were considered to be moderate producers (23.5%), and values higher than 0.32 were strong producers (76.5%). Each bar represents the result of the mean of three biological experiments. Error bars indicate standard deviation.

In this study, the recovered clinical canine *P. aeruginosa* strains were evaluated for their ability to produce biofilm (Figure 2 and Table 4). Among them, 13 (76.5%) were strong biofilm producers, and four (23.5%) resulted to be moderate producers compared to the negative control (Figure 2). Non- or weak biofilm producers were not identified (Figure 2).

Genotypically, the investigated biofilm-encoding genes were detected in the screened strains, as reported in Table 4 and Figure S4, S5, and S6. Precisely, the highest occurrence was recorded for *psl*D detected in all the isolates (100%), followed by *alg*D (94%; 16/17 strains) and *pel*F, which was found only in four strains (23.5%). In particular, four strains classified as strong biofilm producers (ID 6, 7, 8, 9) harbored *psl*D, *alg*D, and *pel*F in combination, while one strong biofilm producer strain (ID 15) was positive only to *psl*D. As reported in Table 4, the majority of the isolated strains (70.6%) carried *psl*D and *alg*D together, but no statistically significant correlation was found between the biofilm producing ability and the presence in combination of *psl*D and *alg*D biofilm-encoding genes (*p* value > 0.5).

The numerical variability of ESBL/MBL genes and the strong or moderate biofilm production in the examined strains did not exhibit a statistically significant relationship (*p* value > 0.5). Furthermore, the simultaneous presence of the tested MBL genes was associated only with two strong biofilm producing strains (ID 8 and 9) which harbored all three biofilm-encoding genes.

## 4. Discussion

*P. aeruginosa* has become a challenging and worrisome pathogen in veterinary medicine, since it is often associated with skin disorders such as chronic otitis externa in pet animals, poorly responding to antimicrobial treatments, due to its resistance to multiple antibiotics [4, 6]. In this study, 17 (28.3%) clinical strains of *P. aeruginosa* were isolated from dogs suffering from chronic otitis externa. Despite the reduced sample size of the study, a statistically significant increase (*p* < 0.05) in the frequency of *P. aeruginosa* isolation associated with canine otitis externa was observed compared to the data from our previous study [1].

The herein strains isolated presented high levels of resistance, especially to β-lactams, such as ceftazidime, imipenem, and meropenem with 100% of the resistant strains, followed by piperacillin-tazobactam (94%) and aztreonam (88%). These obtained results agree with those reported in other studies carried out both in veterinary and human medicine [4, 12, 34–38], suggesting a growing circulation of ESBL- and MBL-producing *P. aeruginosa* strains not only among humans but also in domestic animals and in the environment. Furthermore, it is worth noting that a resistance rate of 94% was also recorded for sulfamethoxazole-trimethoprim. Not belonging to β-lactams, this represents a worrying finding, prospecting an increasingly greater resistance of *P. aeruginosa* to sulphonamides, in accordance with the recent literature. Precisely, a resistance rate of 72.1% to sulfamethoxazole-trimethoprim was observed among *P. aeruginosa* strains isolated from the environment [39], and a 100% resistance level was recorded among canine clinical *P. aeruginosa* isolates reported in a 4-year retrospective study carried out in Italy [4].

Interestingly, herein, all the collected *P. aeruginosa* strains were susceptible to tobramycin and ciprofloxacin, which could be considered a valid therapeutic option to treat infections caused by *P. aeruginosa* strains resistant to β-lactams [40]. In addition, the recovered strains also showed high levels of susceptibility to marbofloxacin (94%), an antibiotic effective for the treatment of otitis, pyoderma, UTIs, and respiratory infections in pet animals, and to gentamicin (82%), often used to topically treat otitis externa in small animals. These comforting results suggest that these two antimicrobial agents can be considered to have potential as antipseudomonal drugs in the treatment of canine skin infections [40, 41].

Nevertheless, it should be considered that all recovered canine *P. aeruginosa* were classified as MDR strains according to Magiorakos et al. [24]. This result agreed with data reported in the literature, which describe an increasing trend in the isolation of MDR *P. aeruginosa* strains in both human and veterinary medicine [4, 6, 38, 42–44]. Multidrug resistance has become one of the greatest public health concerns; as over the years, the abuse and misuse of antimicrobials has led to the selection of pathogenic bacteria showing MDR profiles. In this context, *P. aeruginosa* need special attention, since it is intrinsically resistant to several antimicrobial classes and can acquire resistance to practically any antimicrobial, including ‘last resort' antibiotics such as β-lactams, whose resistance is often mediated by the production of β-lactamases, including ESBLs and MBLs [45].

The phenotypic resistance observed for ceftazidime, imipenem, meropenem, piperacillin-tazobactam, and aztreonam was confirmed genotypically by the presence of ESBL and MBL genes, indicating the production of ESBL and MBL enzymes by the investigated strains. Specifically, the ESBL *bla*_*PER*_ and MBL *bla*_*VIM*_ genes were detected in 100% of the strains. The MBL *bla*_*GES*_ gene was found in 76.5% of the strains, while the ESBL *bla*_*SHV*_ gene was present in 29.4% (5 out 17) of the strains. Additionally, the ESBL *bla*_*TEM*_, the MBL *bla*_*OXA*−48_, and *bla*_*NDM*_ genes were found in 23.5% (4/17) of the strains studied. The lowest prevalence, 17.6% (3/17), was observed for the ESBL *bla*_*CTX*−*M*_ and MBL *bla*_*IMP*_ genes. Our findings differ from those of a 2023 study by Shiralizadeh et al. [12], which identified *bla*_*TEM*_,  *bla*_*PER*_,  *bla*_*OXA*−48_, and *bla*_*IMP*_ as the most isolated genes in *P. aeruginosa* from COVID-19 human patients and did not detect the *bla*_*VIM*_ and *bla*_*GES*_ genes in any of the analyzed strains. Additionally, a study in South Africa by Hosu et al. [36] revealed that *P. aeruginosa* strains yielded in abattoir wastewater and aquatic environment samples were positive only for ESBL *bla*_*TEM*_,  *bla*_*SHV*_, and *bla*_*CTX*−*M*_ genes, which appeared in low percentages in our study. Furthermore, despite some differences, this study aligns with a survey conducted between 2011 and 2018 on *P. aeruginosa* strains from patients of a tertiary-care center in Mexico City, where a high resistance level to carbapenems and monobactams was reported, with *bla*_*VIM*_and *bla*_*GES*_ being the most frequently detected MBL resistance genes, followed by *bla*_*IMP*_ [37].

The presence of ESBL and MBL genes in *P. aeruginosa* isolates from canine otitis externa suggests a significant challenge in treating these infections. Clinical isolates that have acquired ESBLs and MBLs have become a serious problem as carbapenems are one of the last lines of defense against *P. aeruginosa* infections in human medicine. Furthermore, being often located on mobile genetic elements, both ESBL and MBL genes can be easily transferred among bacteria, thus favoring their potential to widespread all over the world [46, 47]. Even though carbapenems, monobactams, and cephalosporins associated with β-lactams are not licensed for companion animals and it is regulated in the EU under the cascade prescribing [22, 48], studies on infection or colonization by ESBL- and MBL-producing *P. aeruginosa* have been reported in animals and animal products [18, 43, 49, 50], thus suggesting the possible anthropogenic origin of ESBL- and MBL-producing *P. aeruginosa* dissemination among companion animals. Indeed, the close contact between animals and humans can facilitate the interspecies transmission of MDR pathogens together with antimicrobial resistance genes, posing a relevant health concern and requiring coordinated efforts in both human and veterinary healthcare to address and mitigate the risks associated with antimicrobial resistance [19, 51, 52].

One of the most challenging traits of *P. aeruginosa* is its capacity to form antibiotic-resistant biofilms, which enhances its resistance and virulence, complicating eradication efforts and increasing the probability of therapeutic failure [53]. In this study, *P. aeruginosa*'s biofilm producing ability was evaluated by the crystal violet-based biofilm assay, and similarly to other studies [38, 54–56], the investigated strains were able to form biofilm, with a large proportion (76.5%) of strong producers. In particular, the present study revealed a high prevalence of *alg*D/*psl*D biofilm genes, which were detected in 70.6% of the strains, both strong and moderate biofilm producers; whilst 4 (23.5%) strains, identified as strong biofilm producers, harbored all three genes *alg*D, *pslD*, and *pel*F. However, in one case, only the *psl*D gene was detected, and the investigated strain resulted to be a strong biofilm producer. The detected biofilm-encoding genes play a key role in biofilm formation: *alg*D, part of the alginate biosynthesis pathway, crucial for the alginate production, is the main component of the biofilm matrix; *pel*F, another important matrix component, is involved in the synthesis of the *Pel* polysaccharide; *psl*D, responsible for the production of the *psl* polysaccharide, plays a critical role in biofilm formation and maintenance. In the context of canine otitis externa, the ability of *P. aeruginosa* to form biofilms is considered a relevant virulence factor, which contributes to the bacterial persistence and resistance, and consequently to chronicity and recurrence of ear infections, as biofilms are more resistant to antibiotic treatment and host immune responses.

However, to the best our knowledge, this is the first investigation carried out to detect ESBL and MBL genes in *P. aeruginosa* of animal origin in Italy, considering its biofilm formation ability.

## 5. Conclusions

This study highlights the critical issue of antimicrobial resistance in *P. aeruginosa* isolates from canine otitis externa. In the last decades, an increased spread of ESBL- and MBL-producing *P. aeruginosa* strains has occurred, reducing the available therapeutic options and leading to worse outcomes for patients. Therefore, the detection of ESBL and MBL genes underscores the importance of ongoing surveillance to prevent and control their spread in veterinary settings. The biofilm formation ability, which is considered a further virulence factor in *P. aeruginosa*, here seemed to show no association with ESBL and MBL gene detection. Given the role of *P. aeruginosa* as a major opportunistic pathogen and its ease of transmission from pet animals to humans or vice versa, ESBL- and MBL-producing *P. aeruginosa* may pose a substantial public health risk.

## Figures and Tables

**Table 1:** Molecular detection of ESBL and MBL genes by PCR.

Gene	Primer sequences (5′-3′)	Annealing temperature	Product size (bp)	References
*bla*_*CTX*−*M*_	F-ATGTGCAGYACCAGTAARGTKATGGC	55	592	[25]
	R-TGGGTRAARTARGTSACCAGAAYSAGCGG			

*bla*_*TEM*_	F-TTTCGTGTCGCCCTTATTCC	60	403	[26]
	R-ATCGTTGTCAGAAGTAAGTTGG			

*bla*_*SHV*_	F-TCAGCGAAAAACACCTTG	52	472	[27]
	R-TCCCGCAGATAAATCACC			

*bla*_*PER*_	F-AATTTGGGCTTAGGGCAGAA	56	925	[28]
	R-ATGAATGTCATTATAAAAGC			

*bla*_*OXA*−48_	F-GCGTGGTTAAGGATGAACAC	58	438	[29]
	R-CATCAAGTTCAACCCAACCG			

*bla*_*IMP*_	F-ACCGCAGCAGAGTCTTTGCC	58	587	[28]
	R-ACAACAAGTTTTGCCTTACC			

*bla*_*VIM*_	F-ATGTTAAAAGTTATTAGTAGT	55	801	[30]
	R-CTACTCGGCGACTGAGCGAT			

*bla*_*NDM*_	F-GGTTTGGCGATCTGGTTTTC	52	621	[31]
	R-CGGAATGGCTCATCACGATC			

*bla*_*GES*_	F-CTGGCAGGGATCGCTCACTC	55	864	[28]
	R-TTCCGATCAGCCACCTCTCA			

**Table 2:** Molecular detection of biofilm-encoding genes.

Gene	Primer sequences (5′-3′)	Amplicon size (bp)	Amplification program
*alg*D	F-CTACATCGAGACCGTCTGCC	593	95°C 5 min; 94°C 30 s, 60°C 40 s, and 72°C 40 s for 30 cycles; 72°C 5 min
	R-GCATCAACGAACCGAGCATC		
*psl*D	F-TGTACACCGTGCTCAACGA	369	
	R-CTTCCGGCCCGATCTTCATC		
*pel*F	F-GAGGTCAGCTACATCCGTCG	789	
	R-TCATGCAATCTCCGTGGCTT		

**Table 3:** Comparison between phenotypic and genotypic resistance results to tested β-lactams.

ID	Phenotypic resistance	ESBL-encoding genes				MBL-encoding genes				
		*bla*_*PER*_	*bla*_*SHV*_	*bla*_*TEM*_	*bla*_*CTX*−*M*_	*bla*_*VIM*_	*bla*_*GES*_	*bla*_*NDM*_	*bla*_*OXA*−48_	*bla*_*IMP*_
1	CAZ, IMI, MRP, SXT	X				X	X			
2	ATM, CAZ, IMI, MRP, SXT, TPZ	X			X	X	X			
3	CAZ, IMI, MRP, SXT, TPZ	X		X	X	X	X			
4	ATM, CAZ, IMI, MRP, SXT, TPZ	X		X		X	X			
5	ATM, CAZ, IMI, MRP, SXT, TPZ	X				X	X			
6	ATM, CAZ, IMI, MRP, SXT, TPZ	X		X		X	X			
7	ATM, CAZ, IMI, MRP, SXT, TPZ	X				X	X		X	
8	ATM, CAZ, IMI, MRP, SXT, TPZ	X	X			X	X	X	X	X
9	ATM, CAZ, IMI, MRP, SXT, TPZ	X	X			X	X	X	X	X
10	ATM, CAZ, IMI, MRP, SXT, TPZ	X	X			X	X			
11	ATM, CAZ, IMI, MRP, SXT, TPZ	X	X			X				
12	ATM, CAZ, IMI, MRP, SXT, TPZ	X				X				
13	ATM, CAZ, IMI, MRP, SXT, TPZ	X				X	X			
14	ATM, CAZ, IMI, MRP, SXT, TPZ	X		X		X				
15	ATM, CAZ, IMI, MRP, SXT, TPZ	X				X		X		X
16	ATM, CAZ, IMI, MRP, SXT, TPZ	X				X	X		X	
17	ATM, CAZ, IMI, MRP, SXT, TPZ	X	X		X	X	X	X		

**Table 4:** Correlation between the biofilm phenotype and the genotype in *P. aeruginosa.*

Biofilm phenotype *n* (%)	Biofilm genotype *n* (%)		
	*psl*D/*alg*D/*pel*F	*psl*D/*alg*D	*psl*D
Strong 13 (76.5%)	4 (30.8%)	8 (61.5%)	1 (7.7%)
Moderate 4 (23.5%)	—	4 (100%)	—
Total 17 (100%)	4 (23.5%)	12 (70.6%)	1 (5.9%)

## Data Availability

The data that represent the findings of this study are available within the article/supporting information.

## Supporting Information

Supporting Information includes Figure S1: PCR for detection of the *bla*_*SHV*_ gene; Figure S2: PCR for detection of *the* *bla*_*TEM*_ gene; Figure S3: PCR for detection of *the* *bla*_*GES*_ gene; Figure S4: PCR for detection of the *alg*D gene; Figure S5: PCR for detection of the *pelF* gene; Figure S6: PCR for detection of the *psl*D gene.
