# Supplementary material for: Detection of Extended-Spectrum β-Lactamases, Metallo-β-Lactamases, Antimicrobial Resistance Profiles, and Biofilm-Forming Capacity in Pseudomonas aeruginosa Strains Recovered From Dogs With Otitis Externa in Italy
Source: Vet Med Int. 2025 May 16;2025:5566151. doi: 10.1155/vmi/5566151 (PMC12101906; doi:10.1155/vmi/5566151)
Supplement: Supporting Information — Additional supporting information can be found online in the Supporting Information section. [file 5566151.f1.pptx]

## Slide 1
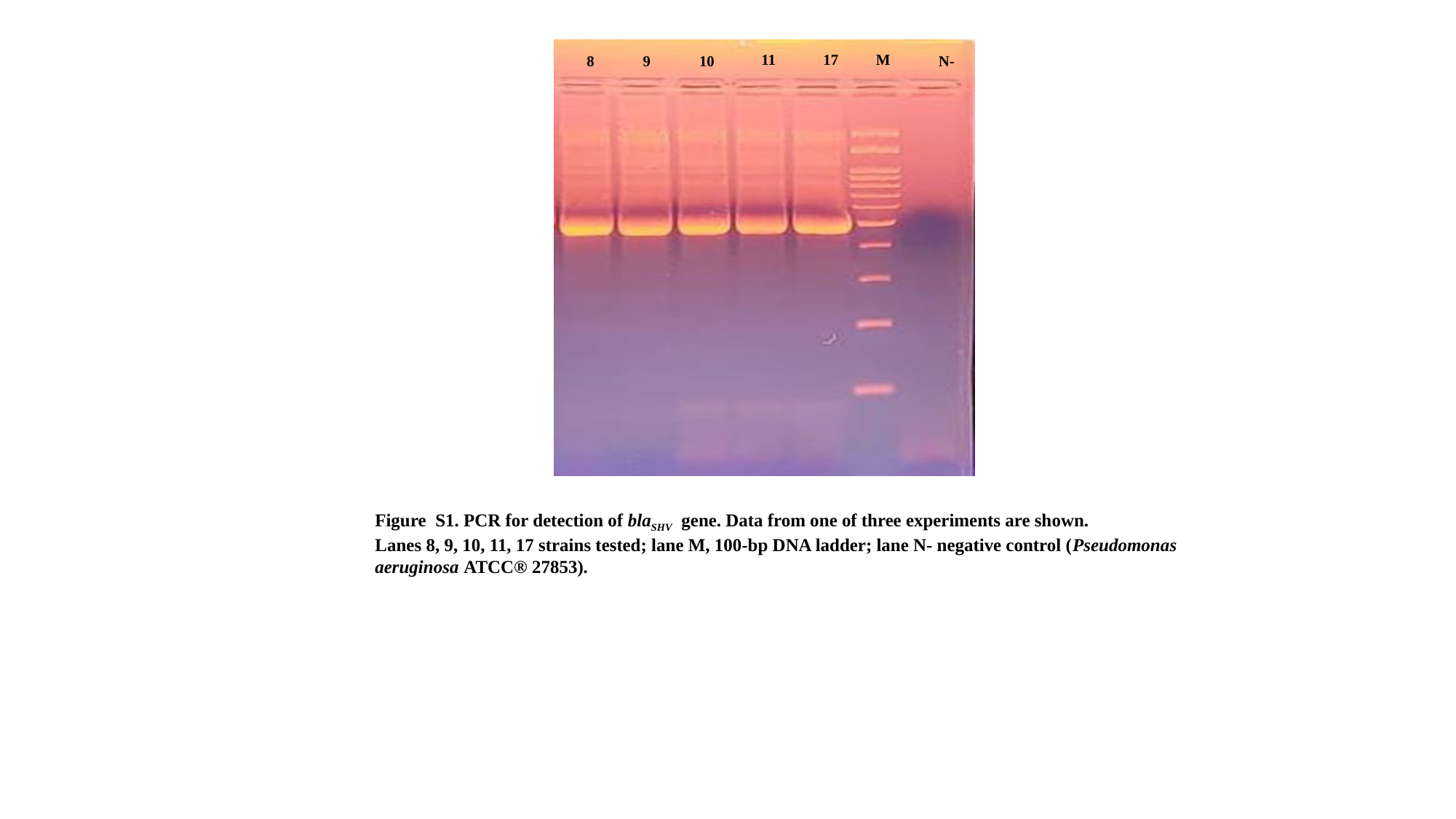

M
11
17
10
N-
8
9
Figure S1. PCR for detection of blaSHV gene. Data from one of three experiments are shown.
Lanes 8, 9, 10, 11, 17 strains tested; lane M, 100-bp DNA ladder; lane N- negative control (Pseudomonas aeruginosa ATCC® 27853).

## Slide 2
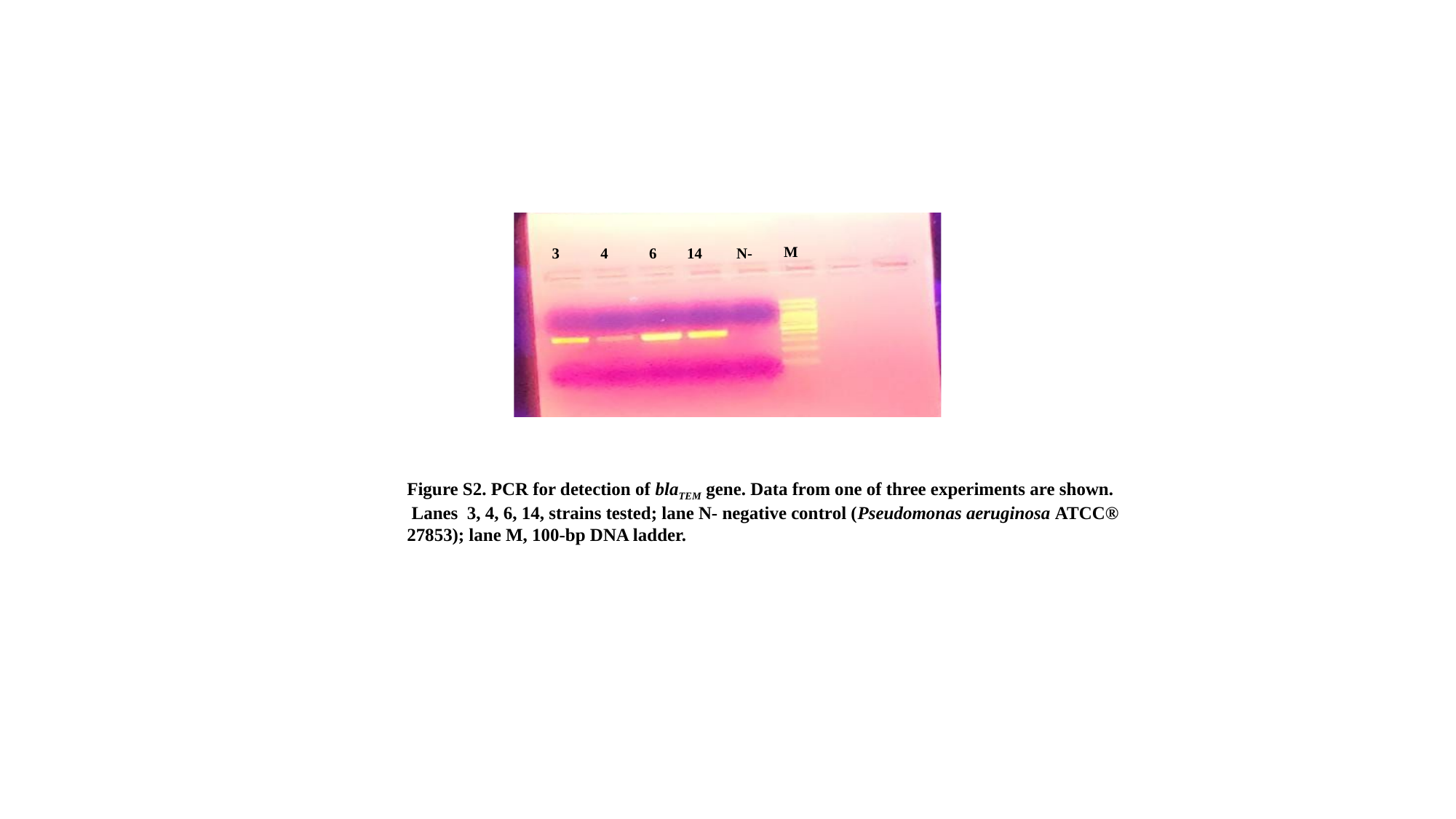

M
N-
14
6
4
3
Figure S2. PCR for detection of blaTEM gene. Data from one of three experiments are shown.
 Lanes 3, 4, 6, 14, strains tested; lane N- negative control (Pseudomonas aeruginosa ATCC® 27853); lane M, 100-bp DNA ladder.

## Slide 3
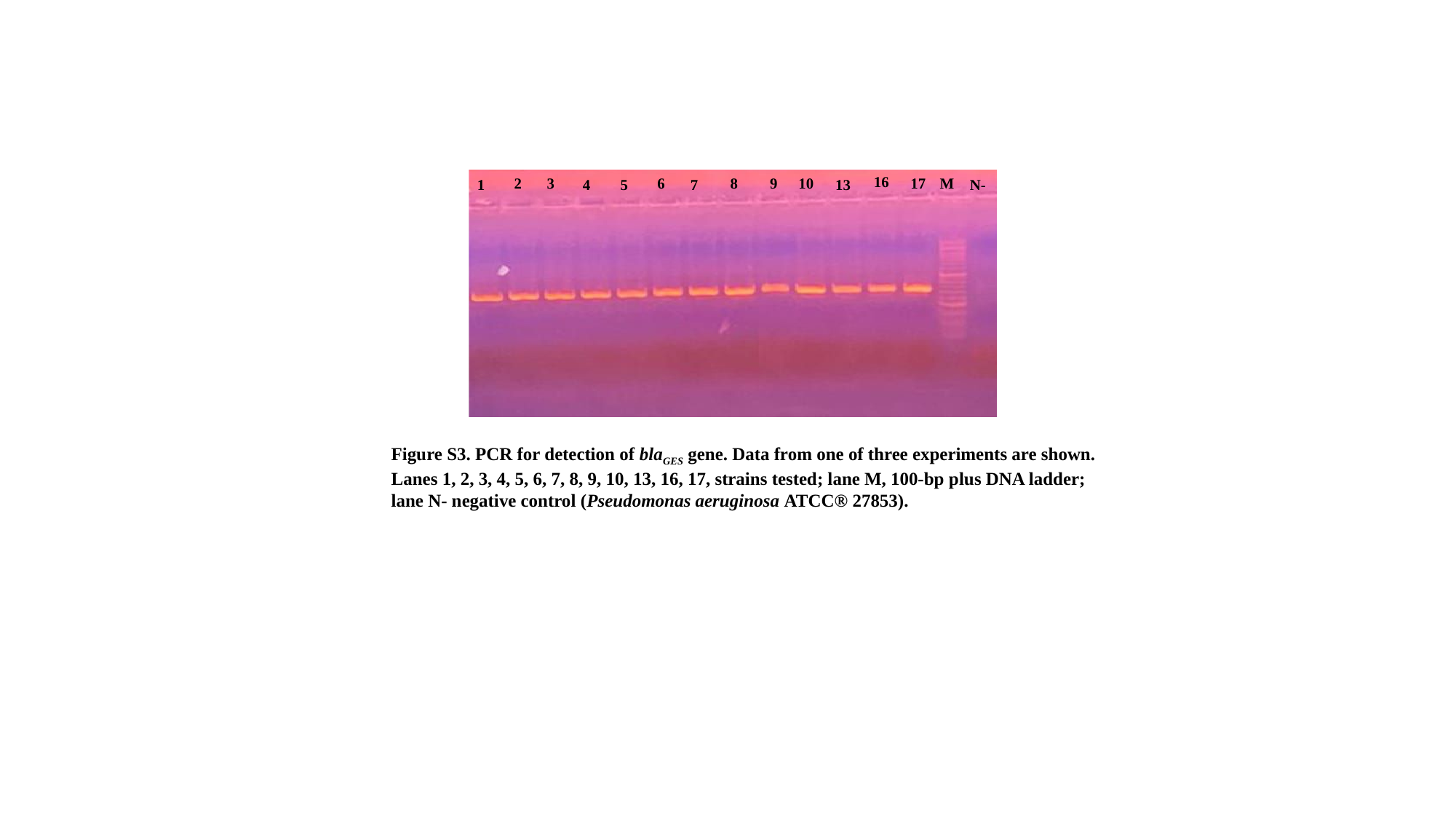

16
M
17
10
9
6
8
2
3
1
4
5
7
N-
13
Figure S3. PCR for detection of blaGES gene. Data from one of three experiments are shown. Lanes 1, 2, 3, 4, 5, 6, 7, 8, 9, 10, 13, 16, 17, strains tested; lane M, 100-bp plus DNA ladder; lane N- negative control (Pseudomonas aeruginosa ATCC® 27853).

## Slide 4
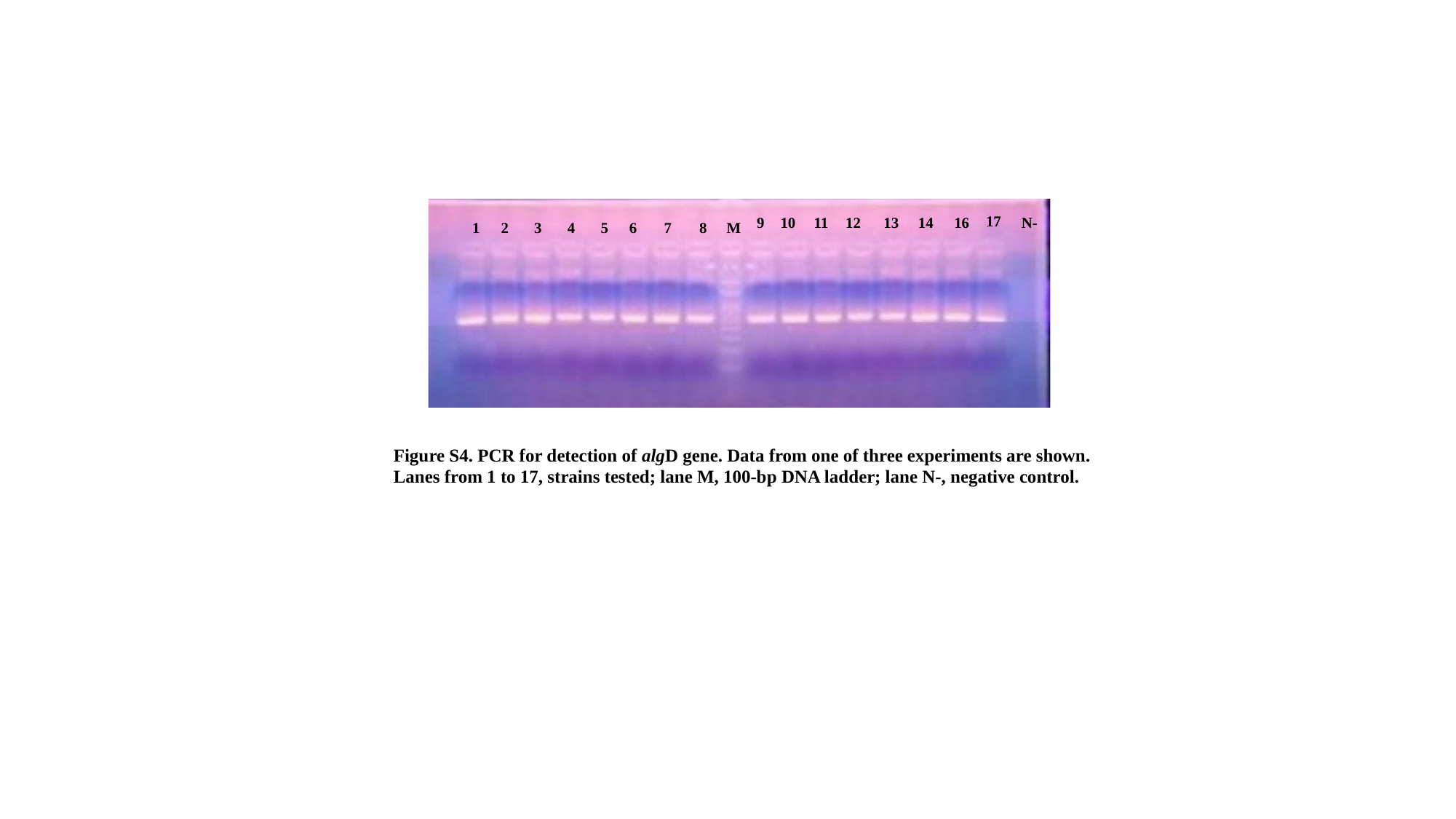

17
N-
14
16
13
12
10
11
9
7
M
8
6
5
4
1
2
3
Figure S4. PCR for detection of algD gene. Data from one of three experiments are shown. Lanes from 1 to 17, strains tested; lane M, 100-bp DNA ladder; lane N-, negative control.

## Slide 5
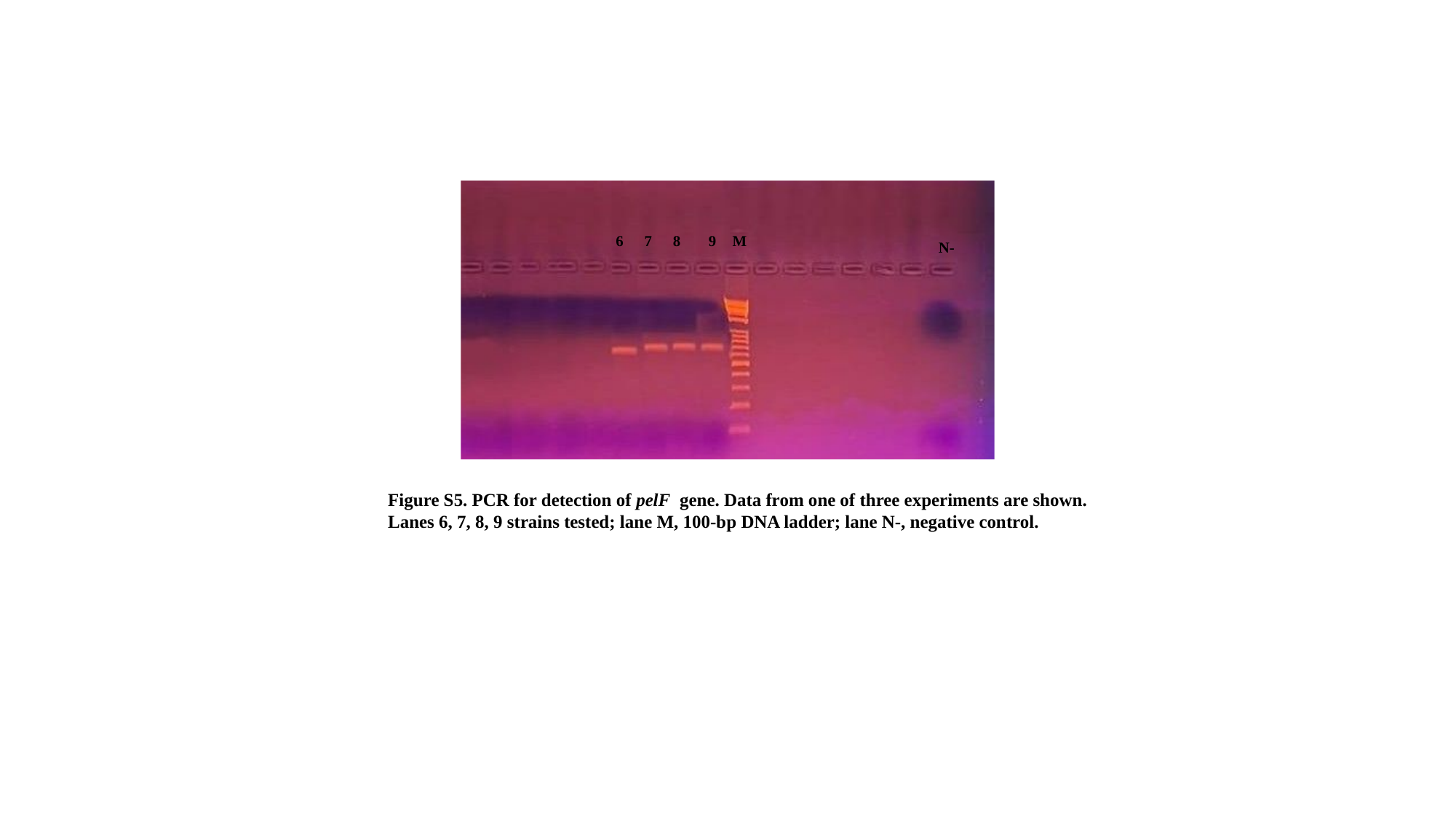

9
M
6
7
8
N-
Figure S5. PCR for detection of pelF gene. Data from one of three experiments are shown. Lanes 6, 7, 8, 9 strains tested; lane M, 100-bp DNA ladder; lane N-, negative control.

## Slide 6
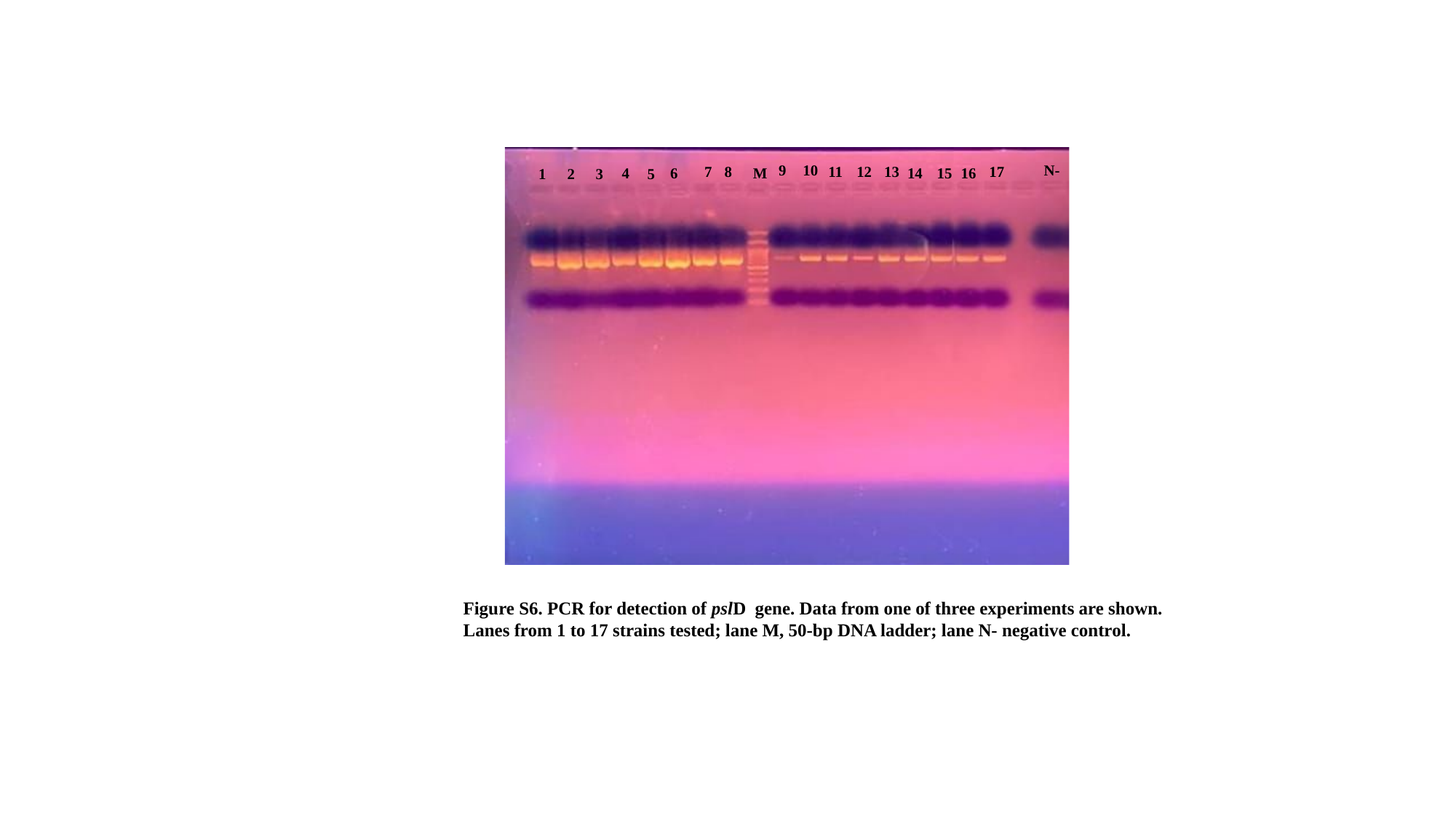

N-
9
10
8
11
12
13
17
7
M
14
15
16
6
4
3
1
2
5
Figure S6. PCR for detection of pslD gene. Data from one of three experiments are shown. Lanes from 1 to 17 strains tested; lane M, 50-bp DNA ladder; lane N- negative control.
